# Children born small-for-gestational-age exhibit normal body fat but elevated hs-CRP and hepatocellular lipid content

**DOI:** 10.1038/s41390-025-04464-5

**Published:** 2025-10-16

**Authors:** Leena Hintikka, Jarmo Jääskeläinen, Riina Palonen, Henrikki Nordman, Raimo Voutilainen, Hanna Huopio, Tomi Laitinen, Juhana Hakumäki

**Affiliations:** 1 Department of Pediatrics, Kuopio University Hospital, Kuopio, Finland; 2 Institute of Clinical Medicine, University of Eastern Finland, Kuopio, Finland; 3 Department of Clinical Radiology, Kuopio University Hospital, Kuopio, Finland; 4 Department of Clinical Physiology and Nuclear Medicine, Kuopio University Hospital, Kuopio, Finland

## Abstract

**Background/objectives:**

Being born small-for-gestational-age (SGA), is associated with an increased risk of cardiometabolic disease in adulthood. We studied the influence of birth size on hepatocellular lipid (HCL) concentrations in prepubertal children. Methods: A total of 195 prepubertal Caucasian children (4.4–9.7 years) were studied in three cohorts. Twenty-one children were born small-for-gestational-age (SGA), 132 were appropriate-for-gestational-age (AGA), and 42 were large-for-gestational-age (LGA). The outcomes were body fat by dual-energy X-ray absorptiometry (DXA), liver fat MRI and MR spectroscopy (MRS), anthropometric measurements at examination, and biochemical markers of metabolism and inflammation.

**Results:**

MRS revealed higher HCL in SGA children, as determined from the methylene (-CH_2_-) resonance (*p* = 0.02). The high-sensitivity c-reactive protein (hs-CRP) level was also significantly higher in the SGA group than in the AGA (*p *= 0.01) or LGA groups (*p *= 0.002). The HCL concentrations were correlated with hs-CRP values in the whole cohort (*R *= 0.51, *p *= 0.03), independently of traditional anthropometric markers. Other laboratory parameters were not associated with HCL.

**Conclusions:**

Our study shows a link between SGA status, elevated hs-CRP, and elevated MRS-detectable HCL. MRS may thus be important in identifying prepubertal children at risk for metabolic sequelae.

**Impact:**

- Prepubertal children born small-for-gestational-age (SGA), although still shorter and leaner than their peers, exhibit elevated liver triglyceride concentrations on proton MR spectroscopy associated with elevated hs-CRP levels.
- Elevated hs-CRP and liver triglyceride concentrations can be early indicators of pathological metabolic changes associated with later cardiometabolic abnormalities.

## Introduction

Several factors during pregnancy may influence fetal development, as well as the individual’s health later in life. Fetal growth restriction prevents the fetus from reaching its genetically determined growth potential.^1^ Being born small-for-gestational-age (SGA) and undernutrition are factors linked to an increased long-term risk of developing insulin resistance (IR), type-2 diabetes (T2D), metabolic syndrome, and cardiovascular diseases.^2,3^ Also being born large-for-gestational-age (LGA) carries similar risks, rendering the relationship between cardiometabolic risks and birth size U-shaped.^4,5^ Epigenetic modifications stimulated by the fetal environment are known to influence gene expression, thus allowing for short- and long-term adaptation to potentially adverse events during development.^6,7^

Anthropometric measurements (e.g., body weight, height, and waist circumference) are commonly used to study children’s growth and body composition. However, visceral fat (VF), which is located in the torso, is more harmful metabolically than subcutaneous fat (SF) and is a particular risk factor for cardiovascular diseases, IR and T2D.^8,9^ Measurements of VF by magnetic resonance imaging (MRI) and computed tomography (CT) are expensive and have limited accessibility, but dual-energy X-ray absorptiometry (DXA) measurements of VF provide an alternative, low-cost technique.^10^ A high correlation between hepatic fat fractions determined using the Dixon MRI method and proton magnetic resonance spectroscopy (MRS) has been demonstrated, but MRI sequences without multifrequency fat modeling lack the robustness necessary for quantifying small fat fractions, as observed in the livers of healthy subjects.^11^ Since MRS has inherently much better spectral resolution than Dixon MRI sequences, it can accurately quantify fat components even at multiple resonance frequencies.

An association between low-grade inflammation and obesity has been convincingly demonstrated in adults and children.^12,13^ Adipose tissue, and VF in particular, is a source of cytokines such as interleukin (IL)-6 and tumor necrosis factor alpha (TNF-α) that drive inflammation by stimulating the hepatic production of acute-phase proteins.^14^ High-sensitivity c-reactive protein (hs-CRP) is a sensitive marker of such inflammation, which in its turn may lead to diabetes, atherosclerosis, and coronary heart disease.^15,16^ SGA children have been found to have higher hs-CRP levels at prepubertal age after adjusting for current BMI.^17^ Inflammation is indeed important in the pathogenesis of childhood metabolic syndrome.^18^ Liver steatosis studies in children have primarily aimed at studying childhood obesity and its adverse metabolic effects. However, an animal study showed that a lack of VF and subcutaneus fat associates with severe IR and accumulation of fat into insulin-sensitive tissues, leading to hepatosteatosis.^19^

In the current study, we investigated the influence of birth size on MRS lipid content, anthropometric measures, and body fat compartments, and cardiometabolic biochemical variables in young children with a median age of 7 years.

## Materials and methods

**Fig. 1:**
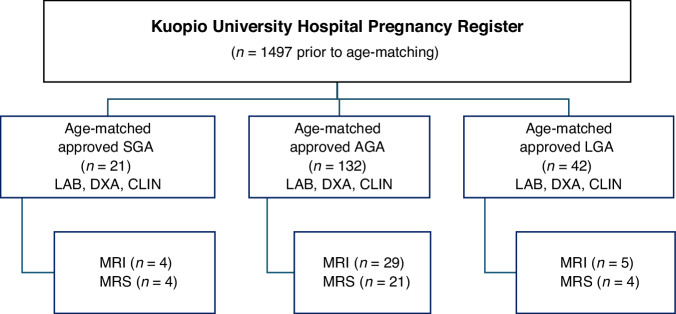
Flowchart of the study subjects. SGA small for gestational age, AGA appropriate for gestational age, LGA large for gestational age, SDS standard deviation score, LAB laboratory test, DXA dual-energy X-ray absorptiometry, CLIN anthropometric measurements at examination, MRI, magnetic resonance imaging, MRS magnetic resonance spectroscopy.

The children were enrolled from the Kuopio University Hospital’s existing clinical pregnancy register according to their birth size and maternal information (Fig. 1). Altogether, 195 children (104 boys) born singleton between 2004 and 2008 were examined at Kuopio University Hospital in Eastern Finland at the age of 4–9 years (median 7.0, interquartile range [IQR] 6.1–7.8 years). This was a cohort study. Birth size was evaluated as SD scores (SDS) for birth weight, length, and head circumference using the current Finnish growth reference.^20^ In this study, SGA was defined as sex-specific birth weight < −2.0 SDS, AGA as birth weight between −2.0 and + 2.0 SDS, and LGA as birth weight > 2.0 SDS.

All participants were contacted by letter and invited to participate in the study. The SGA group included 21 children, the AGA group included 132 children, and the LGA group included 42 children.

MRI was performed on 38 children who could voluntarily participate in imaging without anesthesia. Considering MRS’s special sensitivity, the results for methylene (-CH_2_-) and methyl (-CH_3_) group resonances were determined in 26 children. MRS could be performed only on a small sub-cohort of children, as extremely good cooperation was required due to anesthesia not being allowed in the study.

The exclusion criteria were any continuous medication, a significant developmental delay, a chronic disease other than atopic eczema, allergic rhinitis, or mild asthma requiring no constant medication. Children with acute infection within two weeks before the examination day or with a hs-CRP value > 10 mg/I were excluded from analysis.

Maternal information was obtained from the hospital’s obstetric register and maternity clinic card, including maternal characteristics, medical history, and pregnancy outcomes. Data on background characteristics and lifestyle were also collected using questionnaires and an interview with a trained study nurse.

The children were examined after an overnight (≥10 h) fast. Height was measured using a calibrated Harpenden stadiometer (Holtain Ltd., Crymych, UK) to the nearest millimeter and recorded as the mean of three measurements. Weight was measured using a calibrated digital scale (Seca; Vogel & Halke, Hamburg, Germany) to the nearest 0.1 kg when the participants wore light indoor clothes without shoes. Due to changing body composition and high in childhood and adolescence, the reference values for BMI are age- and sex-specific. Thinness and obesity are defined internationally accepted BMI cutoff points for adult morbidity.^21–23^ BMI was calculated as body weight divided by the square of height (kg/m^2^). SDS for height and BMI was calculated using an adjusted Finnish growth reference, and thinness and significant thinness were defined using BMI SDS cutoff points corresponding to the projected BMI under 17 and 16, respectively, at the age of 18 years.^23,24^ The BMI SDS cutoffs for these categories were −1.65 and −2.22 in girls and −1.83 and −2.35 in boys, respectively. Overweight and obesity were defined using BMI SDS cutoff points corresponding to BMI of 25 and 30, respectively, at the age of 18 years. The BMI SDS cutoffs for these categories were 1.16 and 2.11 in girls and 0.78 and 1.7 in boys, respectively.

Body composition (total fat %) was assessed by DXA using a Lunar device (Lunar Prodigy Advance; GE Medical Systems, Madison, WI) following standard subject positioning and data acquisition protocols. DXA absorptiometry VF was calculated using GE Lunar’s CoreScan application, which utilizes a validated algorithm that estimates the mass of SF and VF in the android region. A geometric and densiometric model was used to subtract off the overlaying SF mass from the total fat mass resulting in a VF mass estimate.^10,25,26^

**Fig. 2:**
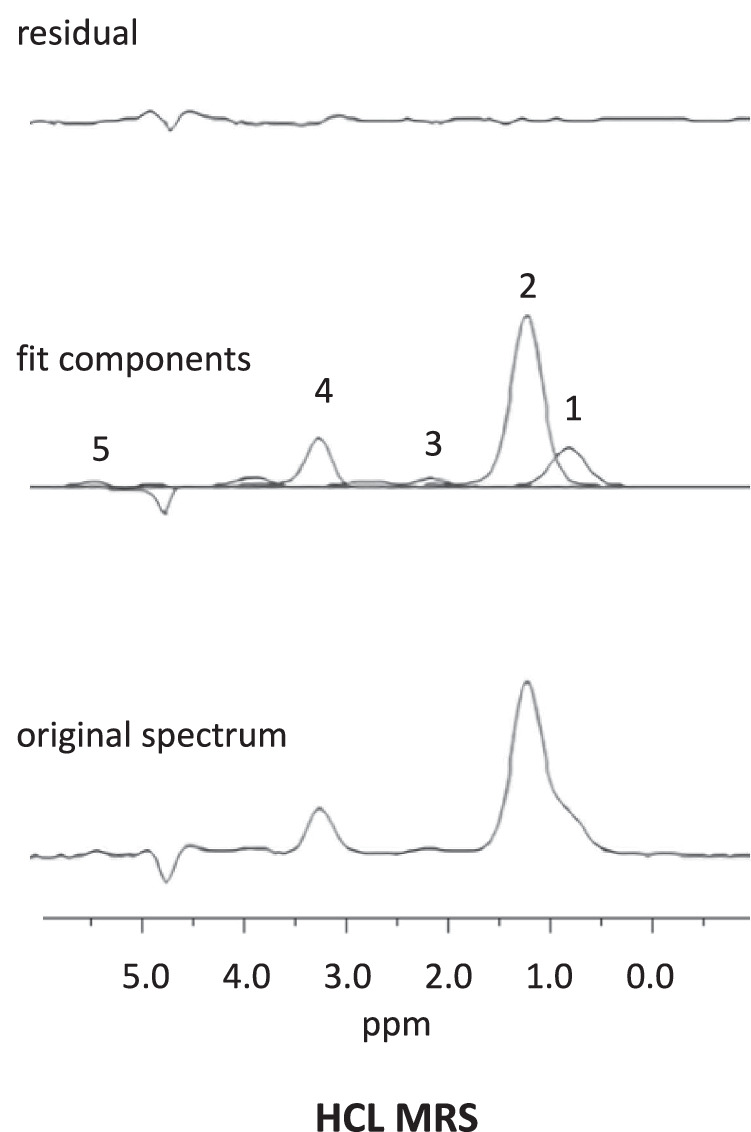
Representative hepatocellular lipid magnetic resonance spectrum (HCL MRS) with the original spectrum, the analyzed fit components, and fit residual. 1 = HCL methyl (-CH_3_) resonance, 2 = HCL methylene (-CH_2_-) resonance, 3 = allylic (-CH_2_-CH = ) resonance, 4 = choline containing compounds (Cho), 5 = vinyl (-CH = CH-) resonance.

MRS and MRI were performed using a 1.5 T MR system (Avanto, Siemens Healthcare, Erlangen, Germany). Hepatocellular lipid (HCL) content was quantified from line-broadened and line-fitted processed spectra as the relation of the intensities of the lipid allylic, methylene (-CH_2_-), and methyl (-CH_3_) group resonances and as internal metabolic control, liver choline-containing compounds (Cho), to the intensity of the water resonance at 4.8 ppm from non-water-suppressed spectra of the same volume of interest according to Machann et al. 2017.^27^ Images of the liver and abdomen were used to position the MRS voxel, avoid blood vessels and gall bladder in the liver, muscle, and bone in the abdomen, and target the voxel into the SF. Data were obtained using a STEAM sequence with a repetition time (TR) of 2000 ms and four signal averages using respiratory triggering for MRS acquisition for HCL and SF. The resonance signal intensities were calculated using AMARES and a Java-based user interface,^28,29^ and a polyunsaturation index (PUI) for SF was calculated according to Machann et al.^27^ A typical liver spectrum is shown in Fig. 2. The results were corrected for differences in T2 and T1 relaxation times of water and fat, except for SF, where a water reference could not be used because of the lack of MRS discernible water in the voxel. By previous literature, we also analyzed the signal percentage of HCL in hepatic water, as described previously.^30^ Respiratory-triggered Dixon-based MRI of the liver and upper abdomen was also obtained and used to calculate the MRI lipid percentage and MRI HCL.^31^

After overnight fasting, blood samples were collected in the morning from the antecubital vein. Blood samples were analyzed in the laboratory at Kuopio University Hospital. The plasma glucose concentration was determined using the hexokinase method (Roche Diagnostics GmbH, Mannheim, Germany). The serum insulin concentration was analyzed using an electrochemiluminescence immunoassay (Roche Diagnostics GmbH). IR was evaluated by using the homeostasis model assessment for insulin resistance (HOMA-IR) as insulin (mU/l) x glucose (mmol/l)/22.5.^32^ According to the International Federation of Clinical Chemistry, the kinetic method was used for plasma alanine transaminase (ALT) concentration measurements (Roche Diagnostics GmbH).

Plasma concentrations of total cholesterol and triglycerides (TG) were analyzed using colorimetric enzymatic assays, and those of high-density lipoprotein cholesterol (HDL-C) and low-density lipoprotein cholesterol (LDL-C) were analyzed using homogeneous colorimetric enzymatic assays (Roche Diagnostics GmbH). Serum hs-CRP was measured by an immunoturbidimetric assay (Roche Diagnostics GmbH).

Data were analyzed using SPSS statistical software version 27 (IBM Corp., Armonk, NY). All data are presented as the median and interquartile range (IQR). To ensure sufficient sample size and to minimize the likelihood of type II error, we performed an a priori power calculation for the primary outcome (at least 0.3 SDS mean difference in BMI SDS) and a post hoc power calculation for MRS analyses to ensure that sufficient sample sizes were reached (using α = 0.05 and power of 0.80).

Statistical significance was set at p-value < 0.05. Analysis of variance (ANOVA) was used to compare the three study groups, SGA, AGA, and LGA, and between each, post hoc test with Bonferroni correction. Analysis of covariance (ANCOVA) was used for variables collected during the clinical examination and adjusted for age and sex unless stated otherwise. Biochemical variables were log-transformed to correct skewed distributions when appropriate. Linear regression analysis evaluated the associations for MRS-derived liver-CH_2_. The model used included birth size group, sex, age, BMI SDS, and hs-CRP as independent variables. The Pearson correlation was performed to evaluate coefficients of hs-CRP with anthropometric measures by birth-weight-group.

### Ethical considerations

The study was conducted in accordance with the Declaration of Helsinki and approved by the Research Ethics Committee of Northern Savo Hospital District (106/2011, 10.2011). All participants and/or their parents gave a written informed consent.

## Results

The background and current offspring characteristics sorted according to birth size are shown in Table 1. At 7 years of age, within the entire study cohort, 3% (6 children) were classified as having significant thinness, 2% (N = 4) as thinness, 65% (N = 126) as normal weight, 18% (N = 35) as overweight and 12% (N = 24) as obese. Median BMI SDS was 0.34. SGA children were shorter and leaner than AGA and LGA children (height and BMI SDS, *p *< 0.05). The LGA children were taller than AGA children (*p *= 0.045), but not heavier (*p *> 0.99), at 7 years. In a sensitivity analysis, using an alternative regrouping of cohorts with definitions SGA <10th percentile and LGA >90th percentile^33^ did not significantly change the sizes of the study groups (SGA children ± 0; AGA –2; LGA + 2), and the interpretation of MRS results remained the same.

**Table 1:** Background, anthropometric, DXA, MRI, MRS and biochemical characteristics.

	SGA	*n*	AGA	*n*	LGA	*n*	*p*
**At birth**							
Gestational age, weeks	39.0 (38.4, 40.6)	21	40.0 (39.0, 40.7)	132	39.8 (38.9, 40.3)	42	0.18
Weight, kg	2.4 (2.2, 2.6)	21	3.6 (3.4, 3.9)	132	4.7 (4.5, 4.9)	42	**<0.001**^**b**^
Weight, SDS	–2.6 (–2.9, –2.3)	21	0.1 (–0.4, 0.7)	132	2.5 (2.2, 2.9)	42	**<0.001**^**b**^
Length, cm	46.0 (45.0, 47.0)	21	50.3 (49.0, 51.0)	132	53.0 (52.0, 54.0)	42	**<0.001**^**b**^
Length, SDS	–2.2 (–2.7, –1.9)	21	–0.2 (–0.6, 0.4)	132	1.4 (1.0, 2.0)	42	**<0.001**^**b**^
**At examination**							
Age, years	6.5 (5.9, 7.2)	21	7.0 (6.0, 7.9)	132	7.1 (6.2, 7.7)	42	0.26
Weight, kg^a^	20.4 (18.8, 23.2)	21	26.4 (22.2, 30.4)	132	28.1 (23.8, 30.3)	42	**0.005**^**c**^
Weight, SDS	–0.8 (–1.2, 0.0]	21	0.6 (–0.4, 1.4)	132	0.3 (–0.3, 1.0)	42	**<0.001**^**c**^
Height, cm^a^	118.4 (114.5, 123.4)	21	125.0 (118.3, 130.1)	132	127.7 (120.9, 131.5)	42	**<0.001**^**c**^
Height, SDS	–0.5 (–1.1, 0.0)	21	0.0 (–0.6, 0.6)	132	0.4 (0.1, 0.9)	42	**<0.001**^**d**^
Waist circumference, cm	51.3. (49.4, 54.0)	21	55.4 (52.8, 62.0)	131	56.8 (53.2, 60.8)	41	**0.04**^**c**^
Waist-to-height ratio	0.44 (0.42, 0.46)	21	0.46 (0.43, 0.49)	131	0.45 (0.43, 0.47)	41	0.10
BMI^a^	14.6 (14.2, 15.7)	21	16.6 (15.3, 19.3)	132	16.6 (15.7, 18.3)	42	**0.004**^**c**^
BMI-SDS	–0.8 (–1.2, 0.0)	21	0.4 (–0.4, 1.3)	132	0.4 (–0.1, 1.2)	42	**<0.001**^**c**^
DXA total body fat, %^a^	17.0 (14.0, 20.2)	21	22.5 (17.6, 30.5)	132	23.4 (16.1, 29.4)	42	**0.01**^**b**^
DXA VF, g^a^	33.0 (24.0, 62.0)	21	63.5 (28.5, 106.0)	132	56.5 (13.8, 94.3)	42	0.39
DXA SF, g^a^	145.0 (81.5, 232.5)	21	259.0 (154.0, 519.0)	131	274.0 (137.5, 383.0)	42	0.08
MRI HCL, %^a^	2.1 (1.8, 4.1)	4	2.9 (2.6, 3.4)	29	2.4 (2.3, 3.8)	5	0.88
MRS HCL, PUI^a^	0.03 (0.02, 0.03)	4	0.04 (0.03, 0.04)	21	0.03 (0.02, 0.05)	4	0.15
MRS HCL -CH_2_-, mM^a^	0.16 (0.08, 0.19)	4	0.08 (0.05, 0.09)	18	0.07 (0.05, 0.09)	4	**0.02**^**c**^
MRS HCL, -CH_3_, mM^a^	0.02 (0.01, 0.02)	4	0.01 (0.1, 0.02)	18	0.01 (0.00, 0.01)	4	**0.04**^**c**^
MRS Cho, mM^a^	0.01 (0.01, 0.01)	4	0.01 (0.01, 0.02)	22	0.01 (0.01, 0.02)	4	0.77
MRS HCL, %,	3.4 (1.5, 3.7)	4	1.1 (0.7, 1.6)	18	0.8 (0.5, 1.4)	4	**0.002**^**c**^
hs-CRP, mg/l^a^	0.6 (0.2, 1.3)	20	0.3 (0.2, 0.7)	132	0.3 (0.1, 0.7)	42	**0.01**^**e**^
Glucose, mmol/l^a^	4.9 (4.6, 5.0)	19	4.9 (4.7, 5.1)	128	4.9 (4.6., 5.1)	42	0.46
Insulin, mU/l^a^	3.9 (3.4, 5.6)	20	4.7 (3.1, 7.0)	132	4.6 (3.5, 6.1)	42	0.86
HOMA-IR	0.9 (0.6, 1.2)	19	1.0 (0.7, 1.5)	128	1.0 (0.7, 1.3)	42	0.42
ALT, U/l^a^	18.0 (13.0, 20.0)	19	16.0 (14.0, 21.0)	132	17.0 (14.0, 23.0)	42	0.60
Total cholesterol, mmol/l^a^	4.4 (4.1, 4.5)	19	4.2 (3.8, 4.7)	132	4.5 (4.0, 4.8)	42	0.72
HDL cholesterol, mmol/l^a^	1.8 (1.6, 2.0)	19	1.6 (1.4, 1.9)	132	1.7 (1.5, 2.0)	42	**0.03**^**f**^
Triglycerides, mmol/l^a^	0.5 (0.5, 0.7)	19	0.6 (0.4, 0.7)	132	0.6 (0.5, 0.7)	42	0.56
LDL cholesterol, mmol/l^a^	2.2 (2.0, 2.6)	19	2.3 (1.9, 2.8)	132	2.5 (2.1, 2.9)	42	0.34

Data are presented as medians (interquartile ranges)—ANOVA between the three study groups.

This study defined SGA as gender-specific birth weight  < −2.0 SDS, AGA as birth weight between −2.0 and + 2.0 SDS, and LGA as birth weight > 2.0 SDS.

*SGA* small for gestational age, *AGA* appropriate for gestational age, *LGA* large for gestational age, *SDS* standard deviation score, BMI body mass index, *DXA* dual-energy X-ray absorptiometry, *VF* visceral fat, *SF* subcutaneous fat, *MRI* magnetic resonance imaging, *HCL* hepatocellular lipid, *MRS* magnetic resonance spectroscopy, *PUI* polyunsaturation index, -*CH*_2_- methylene resonances, *-CH*_3_ methyl resonances, *Cho* liver choline-containing compounds, *mM* millimoles, *hs-CRP* high-sensitivity C-reactive protein, *HOMA-IR* homeostatic model assessment of insulin resistance, *ALT* alanine aminotransferase, *HDL* high-density lipoprotein, *LDL* low-density lipoprotein, *ANOVA* analysis of variance, *ANCOVA* analysis of covariance.

^a^ANCOVA adjusted for the child´s sex and age.

^b^The post hoc test (Bonferroni correction) *P *< 0.001 between SGA and AGA/LGA, AGA and LGA groups.

^c^The post hoc test (Bonferroni correction) *P *< 0.05 between SGA and AGA/LGA groups.

^d^The post hoc test (Bonferroni correction) *P *< 0.001 between SGA and LGA groups, *p *< 0.05 between SGA and AGA groups, AGA and LGA groups.

^e^The post hoc test (Bonferroni correction) *P *< 0.05 between SGA and LGA groups.

^f^The post hoc test (Bonferroni correction) *P *< 0.05 between SGA and AGA groups.

SGA children had a lower total body fat percentage, as measured by DXA, compared to AGA and LGA children (*p *< 0.05), while VF mass was similar across the groups. Measurement of the liver fat fraction using MRS showed a higher -CH_2_- concentration in SGA children (*p *= 0.02, SGA/AGA *p *= 0.01, SGA/LGA *p *= 0.03). Hs-CRP levels were also higher in the SGA children (*p *= 0.01).

**Fig. 3:**
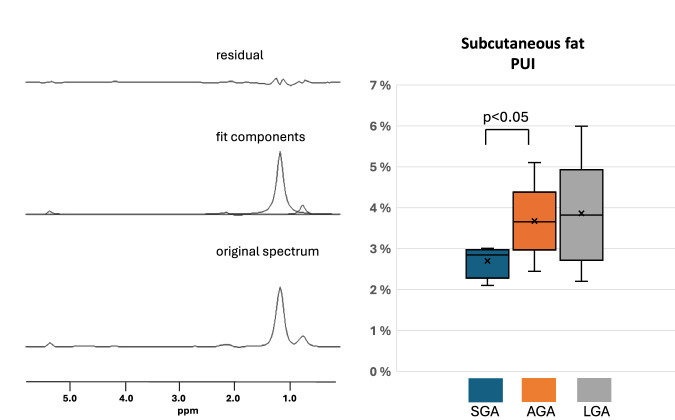
MRS of superficial subcutaneous fat (SF). The polyunsaturation index (PUI) is shown for the three groups of children. SGA children had significantly lower PUI than AGA children did. MRS magnetic resonance spectroscopy, SGA small-for-gestational-age, AGA appropriate-for-gestational-age, LGA large-for-gestational-age.

In a multivariate linear regression analysis, hs-CRP emerged as a significant determinant of the MRS-derived -CH2- peak (*p *= 0.03, Table 2) Unexpectedly, HDL cholesterol levels were also higher in the SGA group (*p *= 0.03, see Table 1). There were no differences in any other biochemical values between the birth size groups. The HCL percentage in the whole study cohort, as determined by MRS was 1.5% ± 1.0% compared to 3.0 ± 1.0% as determined by MRI (difference not significant). We also found that the polyunsaturation index (PUI) of the superficial SF was significantly lower in the SGA group than in the AGA group (Fig. 3).

**Table 2:** Determinants of MRS -CH_2_- (linear regression analysis) in the study cohort.

Independent variables	Standardized B	p (variables)	p (model)	R^2^
Birth size group	−0.18	0.42	0.04	0.29
Male	0.28	0.21		
Age (years) at examination	–0.24	0.22		
BMI-SDS at examination	–0.10	0.61		
hs-CRP, mg/l	0.51	0.03^a^		

*MRS* magnetic resonance spectroscopy, *-CH*_2_*-* lipid allylic methylene group resonances, *SDS* standard deviation score, *BMI* body mass index, *hs-CRP* high-sensitivity C-reactive protein.

^a^Statistically significant dependency, *p* value < 0.05.

The hs-CRP levels showed a significant correlation with BMI SDS, waist-to-height ratio, DXA total body fat %, DXA VF g and DXA SF g parameters in the AGA and LGA subgroups. However, none of these parameters showed a correlation with hs-CRP levels in the SGA group (Table 3).

**Table 3:** The Pearson correlation coefficients of hs-CRP with anthropometric measures and DXA by birth-weight-group.

	hs-CRP SGA	*n*	hs-CRP AGA	*n*	hs-CRP LGA	*n*
BMI-SDS	−0.19	20	0.48^a^	132	0.48^a^	42
Height, SDS	−0.01	20	0.11	132	−0.07	42
Waist-to-height ratio	0.18	20	0.52^a^	131	0.54^a^	41
DXA total body fat, %	0.35	20	0.57^a^	132	0.61^a^	42
DXA VF, g	0.25	20	0.42^a^	132	0.31^b^	42
DXA SF, g	0.34	20	0.44^a^	131	0.66^a^	42

*n* number of individuals, *BMI* body mass index, *SDS* standard deviation score, *DXA* dual-energy X-ray absorptiometry, *VF* visceral fat, *SF* subcutaneous fat, *hs-CRP* high-sensitivity C-reactive protein, *SGA* small for gestational age, *AGA* appropriate for gestational age, *LGA* large for gestational age.

^a^Statistically significant correlation at the p value < 0.01 level.

^b^Statistically significant correlation at the p value < 0.05 level.

## Discussion

This study showed that prepubertal children born SGA present with normal BMI SDS but elevated HCL (i.e. liver triglyceride) concentration on MR spectroscopy, which associates with elevated hs-CRP. Our results suggest lipid accumulation in insulin-sensitive tissues, such as the liver, is possible even in the absence of excess subcutaneous or visceral fat. Lipid accumulation in the liver may stimulate the production of acute-phase proteins, such as hs-CRP, from the liver.^34^ Being born SGA is associated with an increased long-term risk of developing metabolic syndrome.^2,3^ A systematic review showed that the relationship between birth weight and body fat during childhood may be inconsistent. Some studies have suggested that fetally growth-restricted children with increased catch-up growth are at an increased risk of later adiposity, whereas other studies have suggested a neutral or negative association.^35^

Previous studies using proton MR spectroscopy in children,^30^ adolescents,^36,37^ and adults^38^ have shown that elevated levels of liver triglycerides are associated with other metabolic comorbidities, such as higher BMI SDS, higher VF mass, blood fat concentrations, ALT, inflammation, glucose levels, and IR. Body composition varies between sexes and changes remarkably with age.^25^ Metabolic changes, such as the variation of IR between sexes and stages of pubertal development, vary.^38,39^ In this study cohort, we did not observe any other metabolic comorbidities than low grade inflammation, despite elevated liver triglyceride concentration on proton MR spectroscopy. It is noteworthy that this group grew even leaner than AGA controls in the prepubertal period.

Liver biopsy remains the gold standard for assessing the stage of hepatic injury, although MRS is considered a noninvasive imaging standard. In moderately obese children, the assessment of HCL content by MRS has been shown to be more accurate than liver fat volume fraction assessment by abdominal Dixon-based MRI, particularly when hepatic fat fractions are ≤1%.^11^ Based on the MRS-derived triglyceride concentration and liver fat percentage, as determined by MRS, and with a cut-off of 5–5.5%,^30,36,37,40^ the children in our cohort did not present with hepatic steatosis. This is consistent with a previous study on an adult population with no identifiable risk factors for hepatic steatosis.^41^ Interestingly, a recent study in adults suggested that liver fat content exceeding 2% may be considered abnormal if metabolic comorbidities are present.^42^

Obesity in children manifests primarily as increased SF rather than increased intra-abdominal fat. In obese adults, there is a strong correlation between subcutaneous and intra-abdominal fat volumes.^11^ Thus far, limited data are available on the utility of DXA for body composition measurements in prepubertal children,^25,43^ and the results should be interpreted with caution, as many children are thin at this age and the method has been validated for adults only. It is currently believed that accumulated intra-abdominal and hepatic fat are key factors in the development of metabolic disease due to their high endocrine activity and secretion of adipokines, which influence insulin sensitivity and body inflammation. The volume and activity of these fat compartments are not necessarily reflected by classical anthropometric obesity measures, such as BMI. One study in adults indicate that the intra-abdominal fat volume fraction increase more with age than with the degree of obesity. This suggests that short-term overweight primarily leads to an increase in SF, whereas long-term overweight results in increase in intra-abdominal fat.^11^

Interestingly, the SGA group exhibited elevated hs-CRP and HCL levels, independent of BMI SDS. In adults, high hs-CRP levels have been shown to identify non-obese individuals at risk of subclinical atherosclerosis.^44–46^ Furthermore, a meta-analysis has shown that individuals with normal weight can suffer from obesity based on their body fat percentage, which is associated with higher CRP levels;^47^ however, data from non-obese prepubertal children are lacking. However, some biochemical studies have suggested the presence of increased IR and oxidative stress, even in prepubertal normal-weight SGA children.^48,49^ Since CRP is primarily synthesized in liver hepatocytes,^50^ the higher hs-CRP and HCL levels observed in SGA children may indicate a future metabolic risk despite their low BMI SDS and total body fat content. This notion is supported by animal studies, which show that a severe lack of subcutaneous and VF is associated with pronounced IR and fat accumulation in insulin-sensitive tissues, such as the liver.^19^

Interestingly, we also found that the polyunsaturation of SF, as indicated by the PUI,^51^ was significantly lower in SGA children compared to AGA children. In general, SF trends to be more unsaturated than visceral or hepatocellular fat.^27,51,52^ However, it is challenging to draw conclusions from this result, as the signal-to-noise ratio of the HCL data did not permit the calculation of liver PUI, and no supporting literature is available on SF profiles in children. Nonetheless, SF composition and its elevated saturated lipid content may be associated with both IR^53^ and obesity.^52^

The strengths of our study include reliable data on the index pregnancy, such as gestational age and birth weight. Additionally, we had access to extensive anthropometric and biochemical measurements at an early prepubertal age and evaluated overall and central adiposity using DXA and MRS. Our study sample size and participation rate were small but comparable to those of many previous MRS studies on children and adolescents. Although liver biopsy is considered the gold standard for detecting fatty liver disease, we did not obtain biopsy samples due to ethical considerations and the unnecessary risk to patients.

## Conclusions

This preliminary study indicates that prepubertal SGA children may exhibit elevated liver HCL levels despite normal body fat composition. This appears to be accompanied by elevated hs-CRP levels and a reduction in PUI in SF, although a direct causality cannot be claimed at this point. The findings lend support Barker’s hypothesis^2^ regarding the increased risk of metabolic syndrome in SGA children later in adulthood. Furthermore, hs-CRP elevation in SGA children is not detected by traditional anthropometric measures reflecting obesity and adipose tissue accumulation. Thus, our findings suggest that hepatic lipid content at the prepubertal age may serve as an early imaging surrogate for pathological metabolic changes associated with later obesity. Further studies are needed to explore SF and its implications. Although MRS may be challenging to access in public healthcare settings, it remains widely used in academic hospitals worldwide. Thus, we encourage follow-up studies on larger cohorts, as identifying at-risk groups in childhood could enable early obesity interventions before irreversible metabolic changes, such as IR and lipid overflow, occur.

## Data Availability

The datasets presented in this article are available by request after interinstitutional data and material transfer agreement. Requests to access the datasets should be directed to the corresponding author.
